# Supplementary material for: Patient Preferences in Breast Cancer: A Scoping Review
Source: Cancers (Basel). 2025 Dec 31;18(1):134. doi: 10.3390/cancers18010134 (PMC12784654; doi:10.3390/cancers18010134)
Supplement: Supplementary file 1 [file cancers-18-00134-s001.zip › Table S1. search string.pdf]

## Table S1: Search strategy

### ***Database: PubMed (including Medline, via NCBI)***

| Concept | Search string | Number of articles |
|---|---|---|
| **Concept 1: Breast cancer** | "Breast Neoplasms"[Mesh] OR "breast neoplasm"[tiab:~3] OR "breast neoplasm"[tiab:~3] OR "breast neoplasms"[tiab:~3] OR "breasts neoplasms"[tiab:~3] OR "breast neoplasia"[tiab:~3] OR "breasts neoplasia"[tiab:~3] OR "breast tumor"[tiab:~3] OR "breast tumors"[tiab:~3] OR "breast tumour"[tiab:~3] OR "breast tumours"[tiab:~3] OR "breasts tumors"[tiab:~3] OR "breasts tumor"[tiab:~3] OR "breasts tumour"[tiab:~3] OR "breast tumours"[tiab:~3] OR "breast cancer"[tiab:~3] OR "breast cancers"[tiab:~3] OR "breasts cancer"[tiab:~3] OR "breasts cancers"[tiab:~3] OR "breast carcinoma"[tiab:~3] OR "breasts carcinoma"[tiab:~3] OR "breast carcinomas"[tiab:~3] OR "breasts carcinomas"[tiab:~3] OR "mammary cancer"[tiab:~3] OR "mammary cancers"[tiab:~3] OR "mammary carcinoma"[tiab:~3] OR "mammary carcinomas"[tiab:~3] OR "mammary neoplasm"[tiab:~3] OR "mammary neoplasms"[tiab:~3] OR "mammary neoplasia"[tiab:~3] OR "mammary tumor"[tiab:~3] OR "mammary tumors"[tiab:~3] OR "mammary tumours"[tiab:~3] OR "mammary tumour"[tiab:~3] OR "mamma neoplasm"[tiab:~3] OR "mamma neoplasms"[tiab:~3] OR "mamma neoplasia"[tiab:~3] OR "mamma cancer"[tiab:~3] OR "mamma cancers"[tiab:~3] OR "mamma tumor"[tiab:~3] OR "mamma tumors"[tiab:~3] OR "mamma tumour"[tiab:~3] OR "mamma tumours"[tiab:~3] OR "mamma carcinoma"[tiab:~3] OR "mamma carcinomas"[tiab:~3] OR "neoplastic breast"[tiab:~3] OR "neoplastic breasts"[tiab:~3] OR "lobular carcinoma"[tiab:~3] OR "lobular carcinomas"[tiab:~3] OR "lobular tumor"[tiab:~3] OR "lobular tumors"[tiab:~3] OR "lobular tumour"[tiab:~3] OR "lobular tumours"[tiab:~3] OR "lobular neoplasm"[tiab:~3] OR "lobular neoplasms"[tiab:~3] OR "lobular neoplasia"[tiab:~3] OR "ductal carcinoma"[tiab:~3] OR "ductal carcinomas"[tiab:~3] OR "ductal tumor"[tiab:~3] OR "ductal tumors"[tiab:~3] OR "ductal tumour"[tiab:~3] OR "ductal tumours"[tiab:~3] OR "ductal neoplasm"[tiab:~3] OR "ductal neoplasms"[tiab:~3] OR "ductal neoplasia"[tiab:~3] OR "breast sarcoma"[tiab:~3] OR "breast sarcomas"[tiab:~3] OR "breasts sarcoma"[tiab:~3] OR "breasts sarcomas"[tiab:~3] OR "mammary sarcoma"[tiab:~3] OR "mammary sarcomas"[tiab:~3] OR "mamma sarcoma"[tiab:~3] OR "mamma sarcomas"[tiab:~3] | 522.347 articles (09/10/2024; 09:58) |
| **Concept 2: Patient preference** | "Patient Preference"[Mesh] OR "preference*"[tiab] | 214.161 articles (09/10/2024; 09:59) |
| **Concept 1 AND Concept 2** | String concept 1 AND string concept 2 | 3.232 articles (09/10/2024; 10:00) |

## ***Database: Embase***

| Concept | Search string | Number of articles |
|---|---|---|
| **Concept 1: Breast cancer** | ‘breast tumor’/exp OR ((‘breast*’ OR ‘mammary’ OR ‘mamma’ OR ‘lobular’ OR ‘ductal’) NEAR/4 (‘neoplas*’ OR ‘cancer*’ OR ‘tumor*’ OR ‘tumour*’ OR ‘carcinoma*’ OR ‘sarcoma*’)):ti,ab,kw | 825.807 articles (09/10/2024; 10:07) |
| **Concept 2: Patient preference** | 'patient preference'/exp OR ‘preference*':ti,ab,kw | 268.947 articles (09/10/2024; 10:08) |
| **Concept 1 AND Concept 2** | String concept 1 AND string concept 2 | 6.021 articles (09/10/2024; 10:08) |

***Database: CINAHL via EBSCOHOST***

| Concept | Search string | Number of articles |
|---|---|---|
| **Concept 1: Breast cancer** | (MH "Breast Neoplasms+") OR TI ((“breast*” OR “mammary” OR “mamma” OR “lobular” OR “ductal”) N4 (“neoplas*” OR “cancer*” OR “tumor*” OR “tumour*” OR “carcinoma*” OR “sarcoma*”)) OR AB ((“breast*” OR “mammary” OR “mamma” OR “lobular” OR “ductal”) N4 (“neoplas*” OR “cancer*” OR “tumor*” OR “tumour*” OR “carcinoma*” OR “sarcoma*”)) | 129.100 articles (09/10/2024; 10:29) |
| **Concept 2: Patient preference** | (MH "Patient Preference") OR TI ("preference*") OR AB ("preference*") | 53.852 articles (09/102024; 10:34) |
| **Concept 1 AND Concept 2** | String concept 1 AND string concept 2 | 1.254 articles (09/10/2024; 10:35) |

***<u>Database: Web of Science core collections:</u>***

Included editions:

- Science Citation Index Expanded (SCI- EXPANDED)
- Social Sciences Citation Index (SSCI)
- Arts & Humanities Citation Index (AHCI)
- Conference Proceedings Citation Index-Science (CPCI-S)
- Conference Proceedings Citation Index-Social Science & Humanities (CPCI- SSH)
- Book Citation Index-Social Sciences & Humanities (BKCI- SSH)
- Book Citation Index-Science (BKCI-S)
- Emerging Sources Citation Index (ESCI)
- Current Chemical Reactions (CCR- EXPANDED)
- Index Chemicus (IC)

| Concept | Search string | Number of articles |
|---|---|---|
| **Concept 1: Breast cancer** | TS=((“breast*” OR “mammary” OR “mamma” OR “lobular” OR “ductal”) NEAR/4 (“neoplas*” OR “cancer*” OR “tumor*” OR “tumour*” OR “carcinoma*” OR “sarcoma*”)) | 743.766 articles (09/10/2024; 10:15) |
| **Concept 2: Patient preference** | TS=(“preference*”) | 534.206 articles (09/10/2024; 10:16) |
| **Concept 1 AND Concept 2** | String concept 1 AND string concept 2 | 5.225 articles (09/10/2024; 10:17) |

***Database: Scopus***

| Concept | Search string | Number of articles |
|---|---|---|
| **Concept 1: Breast cancer** | TITLE-ABS((“breast*” OR “mammary” OR “mamma” OR “lobular” OR “ductal”) W/4 (“neoplas*” OR “cancer*” OR “tumor*” OR “tumour*” OR “carcinoma*” OR “sarcoma*”)) OR AUTHKEY ((“breast*” OR “mammary” OR “mamma” OR “lobular” OR “ductal”) W/4 (“neoplas*” OR “cancer*” OR “tumor*” OR“tumour*” OR “carcinoma*” OR “sarcoma*”)) | 570. 707 articles (09/10/2024; 10:48) |
| **Concept 2: Patient preference** | TITLE-ABS(preference*) OR AUTHKEY(preference*) | 585.935 articles (09/10:2024; 10:50) |
| **Concept 1 AND Concept 2** | String concept 1 AND string concept 2 | 3.285 articles (09/10/2024; 10:52) |
