# Supplementary material for: Patient Preferences in Breast Cancer: A Scoping Review
Source: Cancers (Basel). 2025 Dec 31;18(1):134. doi: 10.3390/cancers18010134 (PMC12784654; doi:10.3390/cancers18010134)
Supplement: Supplementary file 1 [file cancers-18-00134-s001.zip › Table S2. inclusion_exclusion.pdf]

Table S2: Inclusion and exclusion criteria of the scoping review

Criteria	Inclusion	Exclusion
**Population**	• **Breast cancer patients**<br>• **Breast cancer survivors**	• Healthy people, general community<br>• Other type of cancer patients<br>• Mixed population:<br>○ Breast cancer patients and other type of cancer patients<br>○ Breast cancer patients and healthcare professionals, family members of carers<br>○ Breast cancer patients and the general community<br>• People with a predisposition to breast cancer
**Concept**	• The aim of the study is<br>○ the **elicitation of patient preferences** concerning **treatments and care**	• The aim of the study is<br>○ the identification of patient experiences, opinions, needs – without a ranking or grading of (treatment) elements<br>○ the identification of a choice between treatment and the reasons of the choice – without an assessment of (treatment) elements<br>○ a comparison of two treatments – without an assessment of (treatment) elements<br>• Studies related to<br>○ genetics concerning breast cancer<br>○ fertility<br>○ screening and screening tools<br>○ prevention<br>○ diagnosis and diagnostic tools

		○ shared decision making and patient decision aid development<br><br>○ information and communication needs<br><br>○ treatments not specific for breast cancer<br><br>○ the broader care and follow-up process, including psychological and physical interventions
**Study design**	• The design of the study is a **patient preference study**<br>○ preferences are asked and a ranking/grading of elements is asked<br><br>○ The patient preference study can be<br>▪ Quantitative (e.g., DCE survey)<br><br>AND/OR<br><br>▪ Qualitative (e.g., interviews/ focus groups)	• All other type of study designs, e.g.,<br>○ Reviews<br>○ Retrospective data analysis<br>○ Cost-utility studies<br>○ Clinical trials
**Paper type**		• Full text not available<br><br>• Preprints<br><br>• Conference abstract<br><br>• Conference proceedings<br><br>• Book chapters
**Language**	• Publication in English	• Publications not in English
