# Supplementary material for: Patient Preferences in Breast Cancer: A Scoping Review
Source: Cancers (Basel). 2025 Dec 31;18(1):134. doi: 10.3390/cancers18010134 (PMC12784654; doi:10.3390/cancers18010134)
Supplement: Supplementary file 1 [file cancers-18-00134-s001.zip › Table S3. extraction table template.pdf]

Table S3: Extraction table template

Elements extracted	
General characteristics of the publication	- Title<br>- First author<br>- Country of first author<br>- Year of publication<br>- Journal<br>- Funding
General characteristics patient preference study (PPS)	- Research questions/objectives<br>- Previous steps in study design<br>- PPS method<br>- Ranking/grading method<br>- Conducted by
Population	- Type breast cancer<br>- Breast cancer patients or Breast cancer survivors<br>- Inclusion criteria<br>- Exclusion criteria<br>- Treatments participants<br>- Participants characteristics<br>- Country participants<br>- Sample size<br>- Response rate
	- General information<br>- Ranking/grading elements

Outcomes PPS	- Descriptives elements ranking/grading<br>- Presentation/visualization<br>- Statistic methods<br>- Preference results
Patient heterogeneity	- Assessed?<br>- Assessment methods<br>- Elements/factors
Recruitment strategies & communication	- Recruitment strategy<br>- Compensation participants<br>- Informing and communicating with patients<br>▪ How?<br>▪ When?<br>▪ Who?<br>▪ What?
Patient involvement	- Patient involvement
Evaluation and future research	- Limitations<br>- Strengths<br>- Opportunities
