# Supplementary material for: Patient Preferences in Breast Cancer: A Scoping Review
Source: Cancers (Basel). 2025 Dec 31;18(1):134. doi: 10.3390/cancers18010134 (PMC12784654; doi:10.3390/cancers18010134)
Supplement: Supplementary file 1 [file cancers-18-00134-s001.zip › Table S4. Study descriptives.pdf]

Table S4: Study descriptives

Study	Title	Country of the first author	Journal	Funding	Objectives	PPS method	Ranking/grading elements
Tan et al., 2014	*Assessment of preference for hormonal treatment-related health states among patients with breast cancer*	Singapore	Value in health regional issues	Start-up research grant provided by the National University of Singapore	1) to obtain preference scores for different stages of breast cancer and hormonal therapy–related adverse effects<br>2) to determine associations of patients characteristics (demographic and clinical) and the preference scores	Face-to-face interviews: assessing healthstates using the visual analogous scale (VAS) and the standard gamble method (SG)	- Assessment of 14 health states illustrating<br>○ Common hormonal treatment–associated adverse effects<br>▪ Cataract<br>▪ Hip fracture<br>▪ Wrist fracture<br>▪ Spine fracture<br>▪ Vaginal bleeding<br>▪ Hot flushes<br>▪ Musculoskeletal disorder<br>▪ Pulmonary embolism<br>▪ Endometrial cancer<br>▪ Deep vein thrombosis<br>▪ Ischemic cardiovascular events<br>○ Breast cancer–related disease stages<br>▪ No recurrence<br>▪ Locoregional recurrence<br>▪ Distant recurrence<br>▪ New contralateral breast cancer
Srikanthan et al., 2019	*Assisting with Decision-Making: How Standardized Information Impacts Breast Cancer Patient Decisions Regarding Fertility Trade-Offs and Chemotherapy*	Canada	Journal of adolescent and young adult oncology	Internal departmental funds from Princess Margaret Cancer Centre	1) to evaluate patients' strength of preference for chemotherapy relative to fertility risks , following information delivery<br>2) to evaluate the patient experience during this encounter	Face-to-face interviews: treshold task & postinterview debriefing questionnaire	- Two hypothetical scenarios to assess strenght of preference for chemotherapy<br>○ Treatment schedule<br>○ Follow up<br>○ Short term side effects<br>○ Possible long term side effects<br>○ Survival benefit<br>○ Fertility impact<br>- Chance fertility in the chemotherapy option decreased by 5% intervals → recording treshold point
Silva et al., 2022	*Brazilian breast cancer patient-reported outcomes: What really matters for these women*	Brazil	Frontiers in medical technology	Part of a project that had expenses covered by the Development Impact	1) to identify main relevant outcomes for breast cancer patients in Brazil	Observational descriptive study: exploratory interviews and online questionnaire	- Ranking from least to most important<br>○ Treatment outcomes<br>▪ Adverse effects<br>▪ Pain

				Evaluation – Inter-American Development Bank (DIME-BID)	2) to rank important attributes and outcomes		▪ Quality of life<br>▪ Overall survival<br>▪ Progression free survival<br>○ Impacted aspects of life<br>▪ Social<br>▪ Physical<br>▪ Sexual<br>▪ Economic<br>▪ Psychological and emotional
Ballinger et al., 2017	*Discerning the clinical relevance of biomarkers in early stage breast cancer*	USA	Breast Cancer Research and Treatment	Funding was provided by Susan G. Komen (Grant No. KG090959), and Conquer Cancer Foundation	1) to assess the relative influence of benefit and toxicity risk on chemotherapy selection<br>2) to determine the impact of biomarker information	A choice-based conjoint (CBC) survey	- Pairs of treatment choices varying in<br>○ degree of benefit by risks of recurrence<br>○ toxicity profile, including peripheral neuropathy and congestive heart failure
Wouters et al., 2013	*Disentangling breast cancer patients' perceptions and experiences with regard to endocrine therapy: nature and relevance for non-adherence*	The Netherlands	The Breast	The research presented in this manuscript was financially supported by a grant from the Netherlands Organization for Health Research and Development (ZonMw)	1) to indetify clusters of beliefs and experiences regarding endocrine therapy<br>2) to find potential determinants of non-adherence	Online focus groups and individual interviews with a Q-sorting task	- Sixty-three statements and 11 belief items were used in a Q-sorting task
Hollin et al., 2020	*Do Patient Preferences Align With Value Frameworks? A Discrete-Choice Experiment of Patients With Breast Cancer*	USA	MDM Policy & Practice	/	1) to assess patient preferences for aspects of breast cancer treatments to evaluate and inform the usual assumptions in scoring rubrics for value frameworks	Discrete choice experiment (DCE)	- Minimum life extension for half of patients compared to current therapy<br>- Average increase in toxicity free days compared to current therapy<br>- Changes in major side effects compared to your current therapy<br>- Treatment requirements<br>- Your monthly out-of-pocket costs<br>- Monthly insurance company costs<br>- Available test to see if the therapy will work for you
Smith et al., 2014	*Examining and predicting drug preferences of patients with metastatic breast cancer: using conjoint analysis to examine attributes of paclitaxel and capecitabine*	USA	Breast Cancer Research and Treatment	This study was supported by the Department of Defense Grant	1) to examine variables that influence patients' decisions to undergo treatment for metastatic breast cancer<br>2) to understand the utility of biomarkers in treatment decisions	Online survey containing treatment scenarios for a conjoint analysis	- Method of administration<br>- Likelihood of benefit (defined as shrinkage of advanced cancer, responding to treatment)<br>- Likelihood of a given side effect

Chou et al., 2020	*Health state utilities for metastatic breast cancer in Taiwan*	Taiwan	The Breast	This study was funded by Center for Drug Evaluation, Taiwan	1) to measure health utility values for several metastatic breast cancer (MBC)-related health states and certain breast cancer treatment-related grade 3/4 adverse drug reactions<br>2) to examine whether different methods and respondents' characteristics would influence the utility values elicited	Cross sectional survey: interview with visual analogue scale (VAS) and time trade-off (TTO) to measure health utilities	- Health states including<br>○ Progression-free MBC<br>○ Responding MBC<br>○ Progressive MBC<br>○ Palliative MBC<br>○ Anemia<br>○ Arthralgia<br>○ Diarrhoea<br>○ Fatigue<br>○ Febrile neutropenia<br>○ Nausea/vomiting<br>○ Hand-foot syndrome<br>○ Stomatitis<br>○ Thrombocytopenia
DaCosta DiBonaventura et al., 2014	*Patient Preferences and Treatment Adherence Among Women Diagnosed with Metastatic Breast Cancer*	USA	American Health & Drug Benefits	This study was funded by Eisai Inc	1) to understand how patients trade off medication side effects with improved effectiveness and/or quality of life<br>2) to provide estimates of nonadherence among women with MBC<br>3) to quantify the association of medication nonadherence with health outcomes	Cross sectional internet based survey with a part 'stated preferences and choice task'	- 8 safety attributes<br>○ Alopecia<br>○ Motor neuropathy<br>○ Myalgia/arthralgia<br>○ Nausea/vomiting<br>○ Fatigue<br>○ Neutropenia<br>○ Mucositis/stomatitis<br>○ Diarrhoea<br>- Effectiveness<br>- Dosing regimen<br>- Quality of life
Liu et al., 2024	*Patient preferences and willingness to pay for central venous access devices in breast cancer: A multicenter discrete choice experiment*	China	International Journal of Nursing Studies	None	1) to investigate the preferences of Chinese patients with breast cancer requiring chemotherapy, for central venous access device	Face to face discrete choice experiment (DCE)	- Out of pocket cost<br>- Limitations in activities of daily living<br>- Catheter maintenance frequency<br>- Risk of catheter-related thrombosis<br>- Risk of catheter-related infection<br>- Size of incision
Stamuli et al., 2023	*Patient preferences do matter: a discrete choice experiment conducted with breast cancer patients in six European countries, with latent class analysis*	UK	International Journal of Technology Assessment in Health Care	The study was sponsored by Solid Tumors Franchise of Novartis Oncology Region Europe	1) to examine whether patients' preferences are in line with what is considered important from decision makers viewpoint<br>2) to explore patient preferences with overall survival as an attribute<br>3) to explore whether preferences change depending on how the clinical outcome is measured	Discrete choice experiment (DCE)	- Overall survival<br>- Hyperglycemia<br>- Rash<br>- Pain<br>- Functional well-being<br>- Out-of-pocket payment

Simes et al., 2001	*Patient preferences for adjuvant chemotherapy of early breast cancer: how much benefit is needed?*	Australia	Journal of the National Cancer Institute Monographs	Supported by a grant from the National Health and Medical Research Council, Australia	1) to assess the size of the survival benefit needed to justify the toxicity of chemotherapy, based on the preferences of women who had previously received adjuvant chemotherapy<br>2) to identify patient and disease factors in which larger survival gains would be needed	Semi-structured interviews with time trade-off & survival rate questions	- Survival benefit
Galper et al., 2000	*Patient preferences for axillary dissection in the management of early-stage breast cancer*	USA	Journal of the National Cancer Institute	/	1) to learn more about patients' preferences regarding the trade-offs between the risks and benefits of axillary lymph node dissection (ALND)	Interviews with hypothetical scenarios	- Hypothetical scenarios for four potential benefits<br>○ Local control of the disease<br>○ Survival<br>○ The impact of information obtained from ALND on treatment recommendations<br>○ The prognostic information obtained from ALND independent of its effect on treatment recommendations<br>- against the risk of arm dysfunction
Stamuli et al., 2022	*Patient preferences for breast cancer treatments: a discrete choice experiment in France, Ireland, Poland and Spain*	UK	Future Oncology	The study was sponsored by Solid Tumors Franchise of Novartis Region Europe and received exemption from ethics approval from Western Institutional Review Board (WIRB), an independent research review board, acting at the international level	1) to estimate preferences of breast cancer patients<br>2) to understand breast cancer patients' trade-offs when choosing treatments<br>3) to identify whether patient characteristics can predict treatment choices	Discrete choice experiment (DCE)	- Progression-free survival<br>- Febrile neutropenia<br>- Pain<br>- Functional well-being<br>- Out-of-pocket payment (per year)
Mansfield et al., 2023	*Patient preferences for features of HER2-targeted treatment of advanced or metastatic breast cancer: a discrete-choice experiment study*	USA	Breast Cancer	This study was funded by Daiichi Sankyo, Inc	1) to quantify patients' benefit-risk preferences for attributes associated with HER2-targeted breast cancer treatments<br>2) to estimate minimum acceptable benefits (MABs), denominated in additional months of progression-free survival (PFS), for given treatment-related adverse events (AEs)	Discrete choice experiment (DCE)	- Progression free survival<br>- Nausea/vomiting<br>- Diarrhoea<br>- Liver function problems<br>- Risk of heart failure<br>- Risk of serious lung damage and infections

McQuellon et al., 1995	*Patient preferences for treatment of metastatic breast cancer: a study of women with early-stage breast cancer*	USA	Journal of Clinical Oncology	/	1) to elicit preferences for the treatment of metastatic breast cancer in women with early-stage breast cancer who were given hypothetical treatment scenarios.	Interviews with hypothetical treatment scenarios	- Hypothetical clinical scenarios with side effects varying from low to life-threatening
Spaich et al., 2019	*Patient preferences regarding intraoperative versus external beam radiotherapy for early breast cancer and the impact of socio-demographic factors*	Germany	Archives of Gynecology and Obstetrics	/	1) to explore patient preferences in choosing between intraoperative radiotherapy (IORT) and external beam radiotherapy (EBRT)<br>2) to identify potential factors influencing therapeutic choices	Two part video shown to patients: educational section followed by a preference elicitation section and a questionnaire to identify influencing factors	- Preference elicitation section focusing on additional acceptance risk of recurrence after either treatment
Reinisch et al., 2021	*Patient Preferences: Results of a German Adaptive Choice-Based Conjoint Analysis (Market Research Study Sponsored by Eli Lilly and Company) in Patients on Palliative Treatment for Advanced Breast Cancer*	Germany	Breast Care	This study was sponsored by Lilly Deutschland GmbH	1) to demonstrate the relative importance of overall survival (OS) and progression-free survival (PFS) in relation to quality of life (QoL) and therapy-associated side effects from the perspective of patients with advanced breast cancer	Survey with an adaptive choice- based conjoint measurment	- Therapy goals<br>○ Gained life time<br>○ Gained time without disease progression<br>- QoL<br>○ Emotional balance<br>○ Participation in social life<br>○ Physical agility and mobility<br>○ Flexibility throughout the course of the day/week<br>- Side effects<br>○ Diarrhoea<br>○ Nausea/vomiting<br>○ Hair loss<br>○ Fatigue<br>○ Dry mucosa<br>○ Risk of infection
Ngorsuraches et al., 2015	*Patients' preferences and willingness-to-pay for postmenopausal hormone receptor-positive, HER2-negative advanced breast cancer treatments after failure of standard treatments*	USA	SpringerPlus	Funding from Novartis (Thailand)	1) to understand breast cancer patients' preferences for characteristics of postmenopausal hormone receptor-positive, HER2-negative advanced breast cancer treatments after failure of standard treatments<br>2) to estimate their willingness to pay (WTP)	Discrete choice experiment (DCE)	- Progression free survival<br>- Anemia<br>- Pneumonitis<br>- Monthly treatment cost

Duric et al., 2005	*Patients' preferences for adjuvant chemotherapy in early breast cancer: what makes AC and CMF worthwhile now?*	Australia	Annals of Oncology	This research was supported by grants from the National Health and Medical Research Council of Australia, the Cancer Council of Australia, and the Australia New Zealand Breast Cancer Trials Group	1) to determine the preferences of women treated with adjuvant chemotherapy for early breast cancer by asking the benefits considered necessary to make the treatment worthwhile	Structured, scripted interview using the trade-off method	- Hypothetical scenarios without chemotherapy on<br>○ Life expectancies<br>○ Survival rates
Duric et al., 2005	*Patients' preferences for adjuvant endocrine therapy in early breast cancer: what makes it worthwhile?*	Australia	British Journal of Cancer	This study was initially funded by an NHS South Thames Research and Development Grant (RDP141) and was completed using an unrestricted educational grant from AstraZeneca Pharmaceuticals	1) to determine the preferences of premenopausal women who had adjuvant endocrine therapy in a randomized trial	Semi structured interview with hypothetical clincal scenarios with questions on 'survival time trade-off' and 'survival rate trade-off'	- Hypothetical scenarios without adjuvant endocrine therapy on<br>○ Potential survival times<br>○ Potential survival rates
Omori et al., 2019	*Patients' preferences for postmenopausal hormone receptor-positive, human epidermal growth factor receptor 2-negative advanced breast cancer treatments in Japan*	Japan	Breast Cancer	This study was funded by Eli Lilly Japan K.K.	1) to identify the treatment attributes and their relative importance to patients' treatment preference<br>2) to explore whether patients' sociodemographic and clinical characteristics would affect their preference in their choice of treatment	Discrete choice experiment (DCE)	- Progression free survival<br>- Frequency of stools<br>- Incidence of diarrhoea<br>- Duration of diarrhoea<br>- Route and frequency of administration of the treatment
Nazari et al., 2021	*Preferences of Patients With HR+ and HER2- Breast Cancer Regarding Hormonal and Targeted Therapies in the First Line of Their Metastatic Stage: A Discrete Choice Experiment*	Iran	Value in Health Regional Issues	The authors received no financial support for this research	1) to quantify the preferences of Iranian breast cancer patients regarding the levels of attributes of hypothetical treatment options	Discrete choice experiment (DCE)	- Progression free survival<br>- Stomatitis, grade II & III<br>- Neutropenia, grade III & IV<br>- Arthralgia, grade III & IV<br>- Administration mode<br>- Monthly cost
Kuchuk et al., 2013	*Preference weights for chemotherapy side effects from the perspective of women with breast cancer*	Canada	Breast Cancer Research and Treatment	Funding for this study was received in the form of an unrestricted educational Grant	1) to obtain utility weights from patients with breast cancer for common side effects associated with adjuvant and palliative chemotherapy	Survey with standard gamble questions to obtain preference weights for health states	- Grade I/II (mild to moderate) and III/IV (moderate to severe)<br>○ Diarrhoea<br>○ Hand–foot syndrome<br>○ Mucositis/stomatitis

				from Eisai Pharmaceuticals			○ Nausea<br>○ Sensory neuropathy<br>○ Motor neuropathy<br>○ Fatigue<br>○ Myalgia<br>- Alopecia
Williams et al., 2021	*Quantifying treatment preferences and their association with financial toxicity in women with breast cancer*	USA	Cancer	Financial support for this study was provided by a grant from the Breast Cancer Research Foundation of Alabama	1) to quantify the preferences of patients with breast cancer and their association with financial toxicity	Choice-based conjoint survey design	- Physical side effects<br>- Emotional side effects<br>- Mental side effects<br>- Ability to work<br>- Impact on personal responsibilities<br>- Logistics or convenience<br>- Out-of-pocket events<br>- Impact on activities of daily living<br>- Burden on care partners<br>- Interference with important events<br>- Ability to take part in a clinical trial or use a new medication<br>- Sexual and cosmetic concerns<br>- Fertility
Thill et al., 2016	*Targets for Neoadjuvant Therapy - The Preferences of Patients with Early Breast Cancer*	Germany	GebFra Science	In the preparation of this manuscript the authors received editorial assistance from Dr. Susanne Hell, supported by an unrestricted grant from Roche Pharma AG	1) to investigate which treatment outcomes of a neoadjuvant therapy are considered relevant by patients	Interviews to determine preferences with analytic hierarchy process methods	- Efficacy of the neoadjuvant therapy<br>○ Destruction of tumour cells<br>○ Minimisation of the risk for tumour recurrence<br>○ No reduction of life expectancy due to the disease<br>○ Possibility for breast-preserving operation<br>- Avoidance of side effects of the neoadjuvant therapy<br>○ Side effects that are stressful for the body<br>▪ Fever<br>▪ Diarrhoea<br>▪ Nausea<br>▪ Fatigue<br>○ Side effects that cause bodily changes<br>▪ Loss of hair<br>▪ Weight gain
Wouters et al., 2013	*Trade-off preferences regarding adjuvant endocrine therapy among women with estrogen*	The Netherlands	Annals of Oncology	This work was supported by a grant from the Netherlands Organization for Health Research and	1) to examine breast cancer patients' trade-offs between the benefit and drawbacks of endocrine therapy	Online questionnaire or face- to- face interview: adaptive conjoint analysis choice task	- Efficacy<br>- Libido decrease<br>- Osteoporosis<br>- Hot flashes

	receptor-positive breast cancer			Development (ZonMw) [grant number 152002028]	2) to examine the associations between demographic and clinical characteristics and nonadherence		- Risk of endometrial cancer<br>- Fluid retention<br>- Joint and muscle pain<br>- Regimen duration
Bullen et al., 2024	*Trade-offs between overall survival and side effects in the treatment of metastatic breast cancer: eliciting preferences of patients with primary and metastatic breast cancer using a discrete choice experiment*	UK	BMJ Open	This research was supported by a charitable grant from the Edinburgh and Lothians Health Foundation (Scottish Registered Charity No: SC007342) and was partially supported by the Cancer Research UK (Scotland Centre CTRQQR-2021\100006)	1) to estimate the trade- offs that could be used to inform decision- making at the individual and policy level.<br>2) to investigate minimum acceptable survival (MAS)	Discrete choice experiment (DCE)	- Fatigue<br>- Nausea<br>- Diarrhoea<br>- Additional side effects<br>- Overall survival<br>- Risk of urgent hospital admission
Beusterien et al., 2014	*Use of conjoint analysis to assess breast cancer patient preferences for chemotherapy side effects*	USA	The Oncologist	This work was supported by an unrestricted educational grant from Eisai Pharmaceuticals	1) to evaluate preferences associated with grade I/II and grade III/IV chemotherapy side effects among breast cancer patients receiving chemotherapy<br>2) to assess trade-offs that patients are willing to make between treatment side effects and the route and schedule of treatment administration	Survey with adaptive conjoint analysis to elicit preferences	- Grade I/II and Grade III/IV<br>○ Peripheral neuropathy<br>○ Motor neuropathy<br>○ Myalgia<br>○ Nausea<br>○ Fatigue<br>○ Hand-foot syndrome<br>○ Diarrhoea<br>- Neutropenia<br>- Alopecia<br>- Administration regimen
Thewes et al., 2005	*What survival benefits do premenopausal patients with early breast cancer need to make endocrine therapy worthwhile?*	Australia	The Lancet Oncology	This research was also supported by an unrestricted educational grant-in-aid from AstraZeneca	1) to quantify the survival gains that premenopausal patients with early-stage breast cancer require to justify the side effects and inconvenience of adjuvant endocrine treatments	Face- to- face interview	- Hypothetical clinical scenarios – questions based on<br>○ Improvement of life expectancy<br>○ Improving the probability of survival
Lalla et al., 2014	*Willingness to pay to avoid metastatic breast cancer treatment side effects: results from a conjoint analysis*	USA	SpringerPlus	This research was funded by Genentech, Inc. Xcenda, a consulting company, received funding from Genentech for this study	1) to assess the importance of MBC treatment side effects and to assess the willingness to pay (WTP) to avoid these side effects	Self- administered conjoint analysis survey	- Hair loss<br>- Diarrhoea<br>- Fatigue<br>- Nausea<br>- Tingling in hands and feet<br>- Pain<br>- Risk of infection<br>- Out-of-pocket costs
