# Supplementary material for: Patient Preferences in Breast Cancer: A Scoping Review
Source: Cancers (Basel). 2025 Dec 31;18(1):134. doi: 10.3390/cancers18010134 (PMC12784654; doi:10.3390/cancers18010134)
Supplement: Supplementary file 1 [file cancers-18-00134-s001.zip › Table S5. Population.pdf]

Table S5: Population

Study	Inclusion & exclusion criteria		Study population			
	Inclusion criteria	Exclusion criteria	Breast cancer type	Sample size	Age	Country/Ethnicity
Tan et al., 2014	- Female<br>- Diagnosed with breast cancer<br>- Able to communicate in English or Mandarin<br>- At least 21 years old<br>- Able to function without appartent cognitive impairment	/	Different stages of breast cancer	n = 64<br><br>Response rate: 68.1%	Mean age: 50.1 ± 8.2	Ethnicities involved:<br>Chinese (n=51)<br>Malay (n=7)<br>Indian (n=4)<br>Others (n=2)
Srikanthan et al., 2019	- Female breast cancer survivors<br>- 39 years or younger at diagnosis<br>- Within 2 years of diagnosis<br>- Attended routine outpatient follow-up at a large academic teaching center	- New diagnosis of breast cancer for which they had not received treatment<br>- Within 6 months of diagnosis<br>- Currently undergoing adjuvant chemotherapy<br>- Diagnosed with recurrence<br>- Diagnosis of ductal carcinoma in situ	Breast cancer survivors	n = 50<br><br>Response rate: 86%	Median age: 34.5 (range: 25 – 39)	Recruitment in Canada
Silva et al., 2022	- Brazilian women<br>- Diagnosed with and have been or were being treated for breast cancer	/	Early and metastatic breast cancer	Interviews: n = 12<br><br>Questionnaire: n = 53	Interviews:<br>9 participants age 50 – 69<br>3 participants age 30 – 49<br><br>Questionnaire:<br>age 30 –69	Brazil
Ballinger et al., 2017	- Early stage HER2 negative BC<br>- Received chemotherapy within the prior 8 years	/	Early stage, HER2 negative breast cancer	n = 417	<50: 35%<br><br>> 50: 65%	Ethnicity:<br>Caucasian (88%)<br>African American (5%)<br>Hispanic (4%)<br>Other (3%)
Wouters et al., 2013	/	Patients were excluded for the following reasons:<br>- Metastatic disease<br>- Severe or terminal illness or unclear health status	Early breast cancer	Online focus group n=37<br><br>Q-sorting task	Online focus group<br>Median age: 55<br><br>Q-sorting task	Recruitment in The Netherlands

		- Psychiatric disorder<br>- Regimen completion >1 year ago to avoid recall bias<br>- Language problems		n=14	Median age: 54	
Hollin et al., 2020	- Female<br>- 18 years or older<br>- Self-reported physician diagnosis of stage 3 or stage 4 BC<br>- Diagnosis at least one month before the survey completion<br>- Currently under care of a physician and receiving treatment	- Self-reported diagnosis of rheumatoid arthritis or Alzheimer's disease<br>- Prior to breast cancer diagnosis, a history of other cancers, diseases of blood flow to the brain, chronic kidney disease, chronic obstructive pulmonary disease, coronary artery/heart disease, type 1 or type 2 diabetes, heart failure, hepatitis B, hepatitis C, HIV/AIDS, multiple sclerosis, or tuberculosis	Stage 3 or 4 breast cancer	n = 100	At least 45 years old (72%)	Region (USA):<br>Northeast (n=20)<br>Midwest (n=21)<br>South (n=33)<br>West (n=26)
Smith et al., 2014	- Diagnosed and treated for metastatic breast cancer	/	Metastatic breast cancer	n = 641	Under 40 (13.3%)<br>40-49 (25.9%)<br>50-59 (32.4%)<br>60-69 (24.0%)<br>70-79 (4.1%)<br>80 or over (0.3%)	Caucasian (90.6%)
Chou et al., 2020	- Adult breast cancer patients<br>- 20 years or older<br>- Followed up at the study cancer center of recruitment	- Pregnant<br>- Not able to communicate in Mandarin or Taiwanese Hokkien<br>- Having a cognitive dysfunction	Different stage of breast cancer at diagnosis	n = 102	Mean age: 54.9	Recruitment in Northern Taiwan
DaCosta DiBonaventura et al., 2014	- Diagnosed with metastatic breast cancer<br>- 18 years or older<br>- Proficient in the English language	- Never received treatment with a taxane<br>- Not having health insurance, being covered by Medicaid or not knowing their form of health insurance	Metastatic breast cancer	n = 181<br>Response rate: 7%	Mean age: 52.2	Region of the United States:<br>Midwest (n=51)<br>Northeast (n=37)<br>South (n=48)<br>West (n=44)
Liu et al., 2024	- Being Chinese<br>- ≥18 years	/	Not specified	n = 573	Median age: 50	Recruitment in China

	- Diagnosed with breast cancer by a healthcare professional,<br>- Currently undergoing Chemotherapy					
Stamuli et al., 2023	- 18 years or older<br>- Having breast cancer<br>- From France, Ireland, Poland, Spain and UK	/	Different stages of breast cancer	n = 561	Age range 35 – 44 (28%)	France, Ireland, Poland, Spain, Germany and UK
Simes et al., 2001	- Women who had received at least three cycles of CMF chemotherapy as adjuvant treatment after local treatment for operable breast cancer & who were attending a clinic at the Royal Prince Alfred Hospital, Sydney, Australia, from November 1986 to December 1987<br>- Patients who, having started such therapy, withdrew from it either by their own choice or by the decision of their doctor were also eligible to participate	Exclusion criteria not mentioned; but patients were excluded because:<br>- Insufficient comprehension of English<br>- Too ill to participate<br>- Geographically inaccessible<br>- Died before interview<br>- Were not asked to participate	Different disease statuses at the moment of the interview (disease free, local relapse, distant relapse)	n = 104	Median age: 49	Recruitment in Australia
Galper et al., 2000	- Proficient in spoken and written English<br>*Group 1*: invasive breast cancer & already undergone axillary lymph node dissection (ALDN)<br>- stage I and II breast cancer<br>- completed treatment with breast conserving therapy at least 6 weeks prior to enrolment<br>*Group 2*: women at risk of invasive breast cancer by virtue of a prior ductal carcinoma *in situ* (DCIS)<br>- treated with breast conserving therapy or mastectomy<br>- not undergone ALDN	/	Group 1: invasive breast cancer & already undergone ALDN<br><br>Group 2: women at risk of invasive breast cancer by virtue of a prior DCIS	Group 1: n = 82<br><br>Group 2: n = 62	Group 1: median age: 56 (range: 22 – 80)<br><br>Group 2: median age: 51 (range: 39 – 78)	Recruitment in the USA
Stamuli et al., 2022	- 18 years or older	/	Early stage or advanced/MBC or in remission	n = 371	45 – 54	France, Ireland, Poland and Spain

	- Early stage or advanced/ metastatic breast cancer (MBC) or in remission<br>- Living in one of the four countries of interest					
Mansfield et al., 2023	- Self-reported physician diagnosis of advanced breast cancer (stage III or IV)<br>- 18 years or older<br>- Able to read and understand the language of the study country	/	Advanced/metastatic breast cancer (stage III or IV)	n = 302	Mean age (SD): 47.6 (11.5)	United states, United Kingdom and Japan
McQuellon et al., 1995	- English-speaking patients<br>- Stage 1-IIIa breast cancer<br>- Followed-up in the outpatient Hematology/Oncology clinic at the Comprehensive Cancer Center of Wake Forest University	/	Early stage I-IIIa breast cancer	n = 115	Median age: 57 (range: 32 - 77)	Recruitment in the USA
Spaich et al., 2019	Period of 26 months at the University Medical Centre in Manheim; 101 consecutive patients<br>- with newly diagnosed breast cancer<br>- current candidates for breast-conserving surgery with adjuvant radiotherapy	/	Early breast cancer	n = 101	Mean age: 64 (range: 29 – 89)	Recruitment in Germany
Reinisch et al., 2021	- Female<br>- Postmenopausal<br>- 50 years or older<br>- Locally advanced or metastatic HR+/HER2- breast cancer (de novo or relapsed)<br>- Primary or secondary endocrine resistance<br>- Ongoing systemic treatment (first or second line)	/	HR+/HER2- locally advanced or metastatic breast cancer	n = 104	Mean age: 61	Recruitment in Germany
Ngorsuraches et al., 2015	- 18 years or older<br>- Breast cancer patients at any stage	/	Different stages of breast cancer	n = 146	Average age: 53.2	Recruitment in southern Thailand

	- Having experienced surgery, radiotherapy, chemotherapy, hormonal therapy, or other cancer treatments<br>- Having been in the follow-up period for at least 3 months					
Duric et al., 2005	- Consecutive women who had adjuvant chemotherapy for early breast cancer; 3- 34 month after finishing the treatment	Exclusion criteria not mentioned; but patients were excluded because:<br>- uncontactable or lost to follow-up<br>- inadequate English	Early breast cancer	n = 97	Median age: 55 (range: 25-69)	Recruitment in Australia
Duric et al., 2005	- Women in the Under Fifities Trial from 10 UK hospitals<br>○ invasive, operable early breast cancer<br>○ already been treated with standard surgery<br>○ with or without adjuvant radiotherapy<br>○ with or without<br>○ adjuvant chemotherapy<br>- Completed their adjuvant endocrine therapy 6-30 months previously	/	Early breast cancer	n = 85	Median age: 45 (range: 31-54)	Recruited from the Under Fifities Trial from 10 UK hospitals
Omori et al., 2019	- Postmenopausal women<br>- 45 years or older<br>- HR+ breast cancer	- Pre-menopausal women<br>- Difficulty comprehending the questionnaire (determined by their response to a fixed-choice question)<br>- Withdrew informed consent	Postmenopausal patients with HR+ breast cancer	n = 258	Mean age: 56.7 ± 6.7	Japan
Nazari et al., 2021	- Women older than 18<br>- HR+ breast cancer<br>- Metastatic or non-metastatic	/	HR+ breast cancer (metastatic or non-metastatic)	n = 78	Mean age: 51.35 ± 11.91	Iran

Kuchuk et al., 2013	- Female<br>- Breast cancer, of all stage disease<br>- Currently receiving adjuvant, neoadjuvant, or palliative Chemotherapy<br>- Adequate written and<br>- oral fluency in English<br>- Able to use a compute	/	Different stages of breast cancer	n = 69	Mean age: 54	Recruitment in Canada
Williams et al., 2021	- Women with an early (stage I -III) or a metastatic (stage IV) breast cancer diagnosis<br>- Received services from Patient Advocate Foundation (PAF)	- 18 years or younger<br>- Inability to read English<br>- Lack of a valid email address	Different stages of breast cancer	n = 220<br><br>Response rate: 10%	Median age: 58 (range: 49-66)	Ethnicity:<br>Hispanic or Latino (n=26)<br>Non-Hispanic or Latino (n=194)
Thill et al., 2016	- > 6 months since the primary diagnosis of breast cancer<br>- between > 12 and < 36 months since tumour surgery	/	Early breast cancer	n = 41	For 34 patients; median age: 50	Germany
Wouters et al., 2013	/	- Terminal illness<br>- Psychiatric disorders<br>- Reasons at the discretion of the nurse practitioner or the pharmacist	Estrogen receptor- positive breast cancer	n = 241<br><br>Response rate: 36%	57.2 ± 10	/
Bullen et al., 2024	- Self-identified as having had a primary or metastatic breast cancer diagnosis at some point<br>- Being a UK resident<br>- 18 years or older	/	Different stages of breast cancer	n = 105	/	UK
Beusterien et al., 2014	- Female<br>- Breast cancer patients, with disease of any stage<br>- Currently receiving neo/adjuvant or palliative chemotherapy<br>- Have adequate written and oral fluency in English	/	Different stages of breast cancer	n = 102	Mean age: 54 ± 11.3	Survey implemented in Canada

Thewes et al., 2005	- Diagnosed with early-stage breast cancer 0.5–5.0 years ago<br>- Aged 18–40 years at the time of diagnosis<br>- Adjuvant endocrine therapy for a minimum of 3 months<br>- Have been premenopausal at the time of diagnosis	- Metastatic disease<br>- Not able to give informed consent<br>- Not fluent in English	Premenopausal patients with early- stage breast cancer	n = 102<br><br>Response rate: 75%	36 ± 3 (mean age at diagnosis)	Recruitment in Australia
Lalla et al., 2014	/	/	Metastatic breast cancer	n = 298	< 30: 3 %<br>31-40: 21.8%<br>41-50: 12.1%<br>51-60: 19.5%<br>61-70: 27.5%<br>>70: 16.1%	Region (USA):<br>Northeast (28.9%)<br>Midwest (25.5%)<br>South (23.5%)<br>West (22.1%)
