# Supplementary material for: Patient Preferences in Breast Cancer: A Scoping Review
Source: Cancers (Basel). 2025 Dec 31;18(1):134. doi: 10.3390/cancers18010134 (PMC12784654; doi:10.3390/cancers18010134)
Supplement: Supplementary file 1 [file cancers-18-00134-s001.zip › Table S6. Classification ranking_grading elements.pdf]

Table S6: Classification ranking/grading elements

Categories	*Ranking/grading elements*	*References*
**Adverse events (related to treatments) (n=23)**		[1–3]
	Physical side effects	[4]
	Cataract	[5]
	Hip fracture	[5]
	Wrist fracture	[5]
	Spine fracture	[5]
	Vaginal bleeding	[5]
	Hot flushes	[5,6]
	Musculoskeletal disorder	[5]
	Pulmonary embolism	[5]
	Endometrial cancer	[5]
	Deep vein thrombosis	[5]
	Ischemic cardiovascular events	[5]
	Short term side effects	[7]
	Possible long term side effects	[7]
	Major side effects	[8]
	Pain	[2,9–11]
	Toxicity (profile)	[8,12]
	Anemia	[13,14]
	Arthralgia/Myalgia	[13,15–17]
	Diarrhoea	[11,13,15,17–22]
	Frequency of stools	[23]
	Incidence of diarrhoea	[23]
	Duration of diarrhoea	[23]
	Fatigue	[11,13,15,17,19–22]
	(Febrile) neutropenia	[10,13,15,16]
	Nausea/vomiting	[11,13,15,17–22]
	Hand-foot syndrome	[13,17,22]
	Tingling in hands and feet	[11]
	Mucositis/Stomatitis	[13,15–17]
	Dry mucosa	[19]
	Thrombocytopenia	[13]
	Alopecia	[11,15,17,19,20,22]
	Motor neuropathy	[15,17,22]

	Sensory neuropathy	[17]
	Peripheral neuropathy	[22]
	Hyperglycemia	[9]
	Rash	[9]
	Liver function problems	[18]
	Risk of heart failure	[18]
	Risk of serious lung damage and infections	[18]
	Risk of infection	[11,19]
	Pneumonitis	[14]
	Fever	[20]
	Weight gain	[20]
	Libido decrease	[6]
	Osteoporosis	[6]
	Fluid retention	[6]
	Joint and muscle pain	[6]
**Effectiveness/Efficacy (n=4)**		[1,6,15,20]
**Life expectancies & survival (n=14)**	Survival benefit	[7,9,24]
	Overall survival	[2,9]
	Progression free survival	[2,10,14,16,18,23]
	Life extension	[8]
	Gained life time	[19]
	Gained time without disease progression	[19]
	Life expectancies	[25]
	Improvement of life expectancy	[26]
	(Potential) survival rates	[25,27]
	Potential survival times	[27]
	Improving the probability of survival	[26]
**Quality of life (n=7)**		[2,15]
	Impact on aspects of life – social	[2]
	Impact on aspects of life – physical	[2]
	Impact on aspects of life – sexual	[2]
	Impact on aspects of life – economic	[2]
	Impact on aspects of life – psychological and emotional	[2]
	Limitations in activities of daily living	[28]
	Functional well-being	[9,10]
	Emotional balance	[19]

	Participation in social life	[19]
	Physical agility and mobility	[19]
	Flexibility throughout the course of the day/week	[19]
	Emotional side effects	[4]
	Mental side effects	[4]
	Ability to work	[4]
	Impact on personal responsibilities	[4]
	Logistics or convenience	[4]
	Impact on activities of daily living	[4]
	Interference with important events	[4]
**Administration (n=7)**	Method of administration	[1]
	Administration mode	[16]
	Dosing regimen	[15]
	Treatment schedule	[7]
	Route and frequency of administration of the treatment	[23]
	Regimen duration	[6]
	Administration regimen	[22]
**Breast cancer related disease stages (n=5)**		[5,13]
	Local control of the disease	[24]
	Risk of recurrence	[29]
	Degree of benefit by risk of recurrence	[12]
**Costs (n=8)**	Out-of-pocket costs/Out-of-pocket payments	[8–11,28]
	Out-of-pocket events	[4]
	Insurance company costs	[8]
	Monthly (treatment) cost	[14,16]
**Other (n=6)**		
	Fertility impact	[4,7]
	Treatment requirements	[8]
	Available test to see if the therapy will work	[8]
	Catheter maintenance frequency	[28]
	Risk of catheter-related thrombosis	[28]
	Risk of catheter-related infection	[28]
	Size of incision	[28]
	Information	[24]
	Burden on care partners	[4]
	Ability to take part in a clinical trial or use a new medication	[4]

	Sexual and cosmetic concerns<br>Risk of urgent hospital admission<br>Follow up	[4]<br>[21]<br>[7]

References

1. Smith, M.L.; White, C.B.; Railey, E.; Sledge, G.W. Examining and Predicting Drug Preferences of Patients with Metastatic Breast Cancer: Using Conjoint Analysis to Examine Attributes of Paclitaxel and Capecitabine. Breast Cancer Res. Treat. 2014, 145, 83–89.

2. Silva, A.S.; França, A.C.W.; Padilla, M.P.; Macedo, L.S.; Magliano, C.A.d.S.; Santos, M.d.S. Brazilian Breast Cancer Patient-Reported Outcomes: What Really Matters for These Women. Front. Med. Technol. 2022, 4, 809222.

3. McQuellon, R.P.; Muss, H.B.; Hoffman, S.L.; Russell, G.; Craven, B.; Yellen, S.B. Patient Preferences for Treatment of Metastatic Breast Cancer: A Study of Women with Early-Stage Breast Cancer. J. Clin. Oncol. 1995, 13, 858–868.

4. Williams, C.P.; Gallagher, K.D.; Deehr, K.; Aswani, M.S.; Azuero, A.; Daniel, C.L.; Ford, E.W.; Ingram, S.A.; Balch, A.J.; Rocque, G.B. Quantifying Treatment Preferences and Their Association with Financial Toxicity in Women with Breast Cancer. Cancer 2021, 127, 449–457.

5. Tan, X.Y.; Aung, M.M.; Ngai, M.I.; Xie, F.; Ko, Y. Assessment of Preference for Hormonal Treatment-Related Health States among Patients with Breast Cancer. Value Health Reg. Issues 2014, 3, 27–32.

6. Wouters, H.; Maatman, G.A.; Van Dijk, L.; Bouvy, M.L.; Vree, R.; Van Geffen, E.C.G.; Nortier, J.W.; Stiggelbout, A.M. Trade-Offpreferences Regarding Adjuvant Endocrine Therapy among Women with Estrogen Receptor-Positive Breast Cancer. Ann. Oncol. 2013, 24, 2324–2329.

7. Srikanthan, A.; Amir, E.; Gupta, A.; Baxter, N.; Kennedy, E.D. Assisting with Decision-Making: How Standardized Information Impacts Breast Cancer Patient Decisions Regarding Fertility Trade-Offs and Chemotherapy. J. Adolesc. Young Adult Oncol. 2019, 8, 660–667.

8. Hollin, I.L.; González, J.M.; Buelt, L.; Ciarametaro, M.; Dubois, R.W. Do Patient Preferences Align with Value Frameworks? A Discrete-Choice Experiment of Patients with Breast Cancer. MDM Policy Pract. 2020, 5, 2381468320928012.

9. Stamuli, E.; Corry, S.; Foss, P. Patient Preferences Do Matter: A Discrete Choice Experiment Conducted with Breast Cancer Patients in Six European Countries, with Latent Class Analysis. Int. J. Technol. Assess. Health Care 2023, 39, e21.

10. Stamuli, E.; Corry, S.; Ross, D.; Konstantopoulou, T. Patient Preferences for Breast Cancer Treatments: A Discrete Choice Experiment in France, Ireland, Poland and Spain. Future Oncol. 2022, 18, 1115–1132.

11. Lalla, D.; Carlton, R.; Santos, E.; Bramley, T.; D'Souza, A.Willingness to Pay to Avoid Metastatic Breast Cancer Treatment Side Effects: Results from a Conjoint Analysis. Springerplus 2014, 3, 350.

12. Ballinger, T.J.; Kassem, N.; Shen, F.; Jiang, G.; Smith, M.L.; Railey, E.; Howell, J.; White, C.B.; Schneider, B.P. Discerning the Clinical Relevance of Biomarkers in Early Stage Breast Cancer. Breast Cancer Res. Treat. 2017, 164, 89–97.

13. Chou, T.C.; Chiang, S.C.; Ko, Y. Health State Utilities for Metastatic Breast Cancer in Taiwan. Breast 2020, 51, 57–64.

14. Ngorsuraches, S.; Thongkeaw, K. Patients' Preferences and Willingness-to-Pay for Postmenopausal Hormone Receptor-Positive, HER2-Negative Advanced Breast Cancer Treatments after Failure of Standard Treatments. Springerplus 2015, 4, 674.

15. DiBonaventura, M.D.; Copher, R.; Basurto, E.; Faria, C.; Lorenzo, R. Patient Preferences and Treatment Adherence Among Women Diagnosed with Metastatic Breast Cancer. Am. Health Drug Benefits 2014, 7, 386.

16. Nazari, A.; Lopez-Valcarcel, B.G.; Najafi, S. Preferences of Patients With HR+ & HER2- Breast Cancer Regarding Hormonal and Targeted Therapies in the First Line of Their Metastatic Stage: A Discrete Choice Experiment. Value Health Reg. Issues 2021, 25, 7–14.

17. Kuchuk, I.; Bouganim, N.; Beusterien, K.; Grinspan, J.; Vandermeer, L.; Gertler, S.; Dent, S.F.; Song, X.; Segal, R.; Mazzarello, S.; et al. PreferenceWeights for Chemotherapy Side Effects from the Perspective ofWomen with Breast Cancer. Breast Cancer Res. Treat. 2013, 142, 101–107.

18. Mansfield, C.; Botha, W.; Vondeling, G.T.; Klein, K.; Wang, K.; Singh, J.; Hackshaw, M.D. Patient Preferences for Features of HER2-Targeted Treatment of Advanced or

Metastatic Breast Cancer: A Discrete-Choice Experiment Study. Breast Cancer 2023, 30, 23–35.

19. Reinisch, M.; Marschner, N.; Otto, T.; Korfel, A.; Stoffregen, C.; Wockel, A. Patient Preferences: Results of a German Adaptive Choice-Based Conjoint Analysis (Market Research Study Sponsored by Eli Lilly and Company) in Patients on Palliative Treatment for Advanced Breast Cancer. Breast Care **2021**, 16, 491–499.

20. Thill, M.; Pisa, G.; Isbary, G. Targets for Neoadjuvant Therapy–The Preferences of Patients with Early Breast Cancer. Geburtshilfe Frauenheilkd 2016, 76, 551–556.

21. Bullen, A.; Ryan, M.; Ennis, H.; Gray, E.; Loria-Rebolledo, L.E.; McIntyre, M.; Hall, P. Trade-Offs between Overall Survival and Side Effects in the Treatment of Metastatic Breast Cancer: Eliciting Preferences of Patients with Primary and Metastatic Breast Cancer Using a Discrete Choice Experiment. BMJ Open 2024, 14, e076798.

22. Beusterien, K.; Grinspan, J.; Kuchuk, I.; Mazzarello, S.; Dent, S.; Gertler, S.; Bouganim, N.; Vandermeer, L.; Clemons, M. Use of Conjoint Analysis to Assess Breast Cancer Patient Preferences for Chemotherapy Side Effects. Oncologist 2014, 19, 127–134.

23. Omori, Y.; Enatsu, S.; Cai, Z.; Ishiguro, H. Patients' Preferences for Postmenopausal Hormone Receptor-Positive, Human Epidermal Growth Factor Receptor 2-Negative Advanced Breast Cancer Treatments in Japan. Breast Cancer 2019, 26, 652–662.

24. Galper, S.R.; Lee, S.J.; Tao, M.L.; Troyan, S.; Kaelin, C.M.; Harris, J.R.; Weeks, J.C. Patient Preferences for Axillary Dissection in the Management of Early-Stage Breast Cancer. J. Natl. Cancer Inst. 2000, 92, 1681–1687.

25. Duric, V.M.; Stockler, M.R.; Heritier, S.; Boyle, F.; Beith, J.; Sullivan, A.; Wilcken, N.; Coates, A.S.; Simes, R.J. Patients' Preferences for Adjuvant Chemotherapy in Early Breast Cancer: What Makes AC and CMF Worthwhile Now? Ann. Oncol. 2005, 16, 1786–1794.

26. Thewes, B.; Meiser, B.; Duric, V.M.; Stockler, M.R.; Taylor, A.; Stuart-Harris, R.; Links, M.; Wilcken, N.; McLachlan, S.A.; Phillips, K.A.; et al. What Survival Benefits Do Premenopausal Patients with Early Breast Cancer Need to Make Endocrine Therapy Worthwhile? Lancet Oncol. 2005, 6, 581–588.

27. Duric, V.M.; Fallowfield, L.J.; Saunders, C.; Houghton, J.; Coates, A.S.; Stockler, M.R. Patients' Preferences for Adjuvant Endocrine Therapy in Early Breast Cancer: What Makes It Worthwhile? Br. J. Cancer 2005, 93, 1319–1323.

28. Liu, S.; Xiang, Y.; Gu, Y.; Chen, N.; Fu, P.; Wei, Y.; Zhao, P.; Li, Y.; Du, C.; Mu, W.; et al. Patient Preferences and Willingness to Pay for Central Venous Access Devices in Breast Cancer: A Multicenter Discrete Choice Experiment. Int. J. Nurs. Stud. **2024**, 152, 104695.

29. Spaich, S.; Krickeberg, S.; Hetjens, S.;Wenz, F.; Gerhardt, A.; Sutterlin, M. Patient Preferences Regarding Intraoperative versus External Beam Radiotherapy for Early Breast Cancer and the Impact of Socio-Demographic Factors. Arch. Gynecol. Obstet. 2019, 299, 1121–1130.
