# Supplementary material for: Patient Preferences in Breast Cancer: A Scoping Review
Source: Cancers (Basel). 2025 Dec 31;18(1):134. doi: 10.3390/cancers18010134 (PMC12784654; doi:10.3390/cancers18010134)
Supplement: Supplementary file 1 [file cancers-18-00134-s001.zip › Table S7. preference outcomes.pdf]

Table S7: Preference outcomes

Study	Preference results		Prefererence heterogeneity		
	(Statistic) method	Results	Association assessed	Assessment methods	Results
Tan et al., 2014	- Preference scores obtained for each health state; presented by means, medians, and SDs	➢ Health states with **locoregional and distant recurrence obtained lower preference scores** than "no recurrence" health states<br>➢ **Distant recurrence** health states were given **lower preference scores**<br>➢ Adverse effects such as **ischemic cerebrovascular events** and **spine fracture** – lowest preference scores	- Association **patients characteristics** and preference scores	- Spearman's correlation coefficient, Mann-Whitney U test, Kruskal-Wallis test	➢ **Ethnicity:** Chinese patients reported lower Visual Analogue Scale (VAS) scores for 'distant recurrence with chemotherapy adverse effects' compared to Malay patients<br>➢ **Education level**: Patients with higher education levels had higher Standard Gamble (SG) scores for adverse effects (wrist fracture, vaginal bleeding) than those with lower education levels<br>➢ **Age**: Age was positively correlated with VAS scores for cataracts, deep vein thrombosis, and new contralateral breast cancer, but negatively associated with SG scores for wrist fractures and ischemic cerebrovascular events<br>➢ **Language version**: Respondents selecting the Chinese version of the questionnaire had lower SG and VAS scores for 'distant recurrence with chemotherapy adverse effects', as well as lower SG scores for spine fracture and endometrial cancer
Srikanthan et al., 2019	- Threshold task	➢ Most women **not willing to trade-off survival benefits** of adjuvant therapy **to maintain fertility**	- **Independent variables** (age, marital status, parity at diagnosis, education, financial role, and household income) as predictors for **willingness to accept a reduction in fertility**	- Univariable logistic regression	➢ Demonstrated **no significant predictors** for willingness to accept reduction in fertility
Silva et al., 2022	- Observational study: exploratory interviews & online questionnaire (classify and rank)	➢ **Psychological, emotional and sexual impacts** frequently described<br>➢ Order of importance ranking: 1) overall survival, 2) progression-free survival, 3) quality of life	/	/	/
Ballinger et al., 2017	- Choice-based conjoint analysis	➢ Greatest share of preference (39%) for the risk/benefit profile most similar to the non-anthracycline regimen<br>○ Largest shifts in preferences with **alterations in risk reduction benefit** & **peripheral neuropathy likelihood**	- Perceived risk of recurrence, prior treatment setting, survey group, prior experience with peripheral neuropathy (PN)	- Subgroup analysis	➢ **Perceived risk of recurrence**:<br>○ *Perceived low risk* → preferred a non-anthracycline regimen<br>○ *Higher perceived risk* → more inclined toward an anthracycline plus taxane regimen<br>➢ **Prior experience with PN**:<br>○ *Previous PN*

					→ slightly stronger preference for taxane-containing regimens with moderate PN risk<br>→ significantly lower preference for a non-taxane-containing regimen with zero risk of PN
Wouters et al., 2013	- Hierarchical cluster analysis	➢ Six cluster solution: 1) information, 2) efficacy, 3) tenacity, 4) coping, 5) side effects, 6) usage<br>➢ Most important: experiences and beliefs with regard to 'information', 'tenacity' and 'coping'	/	/	/
Hollin et al., 2020	- Logit-based regression models; preference weights for each attribute level	➢ Better clinical outcomes → higher preference weights<br>➢ Most important: **life extensions**<br>➢ Other important attributes: **out-of-pocket cost of treatment**, **treatment route of administration**, **availability of reliable tests** to help gauge treatment efficacy	- Only cancer stage was correlated with class assignment – late-stage patients (stage 4) more likely represented by preferences in Class 2	- Latent class logit model	➢ **Class 1 preferences:** treatment out-of-pocket cost - most important<br>➢ **Class 2 preferences:** treatment efficacy - most important
Smith et al., 2014	- Conjoint analysis; Utility values – numeric representation of a preference	➢ Treatment associated with greater likelihood of benefit or lower toxicity: higher response rate<br>➢ **More sensitive for benefit**	- Respondent age, presence and age of children, proximity to treatment site, prior chemotherapy experience	- Subgroup analysis	➢ **Age:** modest but statistically significant effect on treatment choice<br>o Respondents under 50: more likely to opt for treatment, regardless of benefit or toxicity levels<br>o With oral administration and severe diarrhoea, significant age group differences were observed at 30% and 50% benefit, but not at 20%.<br>o At 27% benefit and oral administration, significant differences between age groups were found only in cases of severe, not moderate, toxicity<br>➢ **Children:**<br>o respondents with children under 18 → more likely to choose treatment, with an even higher likelihood among those with children under 12<br>o No significant differences were found between respondents with adult children and those without children<br>➢ **Distance to treatment site**:<br>o Respondents with a travel time of less than 30 minutes to the treatment site → significantly more likely to opt for treatment compared to those facing longer travel times<br>➢ **Prior chemotherapy experience**:<br>o Respondents with prior capecitabine use → more likely to choose a drug with a similar capecitabine profile, while those with prior taxane use were more likely to opt for a drug resembling paclitaxel

Chou et al., 2020	- Scores transformed into utility values	➢ Most preferred state: "responding MBC"<br>➢ Most acceptable adverse drug reaction (ADR): fatigue<br>➢ Least acceptable ADR: nausea/vomiting	- Comparison of utility values for the four MBC health states among various demograic subgroups (age, educational level, marital status, income, DNR (do not resuscitate) status)	- Spearman's test, Mann-Whitney U test, Kruskal-Wallis test	➢ **Age:** negatively correlated with utility values for 'progression-free MBC'<br>➢ **Education level:** Respondents with higher education levels → higher utility values, with statistical significance found only for 'progression-free MBC' and 'palliative MBC'<br>➢ **Household income**: utility values were lowest among those with a household income of 30,000 per month<br>➢ **Marital status:** Married respondents had higher utility values across all metastatic breast cancer states compared to those who were not married<br>➢ **Signed the DNR**: Patients who signed a DNR order → lower utility values in all metastatic breast cancer health states, with a significant difference observed only in the 'progressive MBC' state
DaCosta DiBonaventura et al., 2014	- Hierarchical Bayesian logistic regression models to analyze patient preferences	➢ Effectiveness (overall survival): primary importance; followed by side effects (alopecia, fatigue, neutropenia, motor neuropathy, nausea/vomiting)	/	/	/
Liu et al., 2024	- Conditional logit model & mixed logit model	➢ **Catheter maintenace frequency**: strongly favouring **one time a month**<br>➢ Lowest risk of catheter-related trombosis & lowest risk of catheter-related infection: strong preference	- Based on distance, age, medical insurance type, geographic region	- Subgroup analysis	➢ **Distance:** Patients who had to travel over 1 hour to the hospital → significantly more willing to pay for reduced catheter maintenance frequency than those with travel times under 1 hour<br>➢ **Age:** Patients under 50 → more willing to pay for a central venous access device that does not affect their daily activities compared to those over 50<br>➢ **Medical insurance:** Patients with Urban Employee Basic Medical Insurance (UEBMI) → greater concern about almost all attributes compared to those with Urban and Rural Resident Medical Insurance (URRMI)<br>➢ **Geographic region:** Patients from the eastern region → willing to pay more to reduce the risk of catheter-related infections and thrombosis compared to patients from western or central regions
Stamuli et al., 2023	- Heteroscedastic conditional logit model & Mixed logit models	➢ Patients are willing to pay the largest amount of out-of-pocket payment (OOP) for i) avoiding levels of pain, ii) functional well being (FWB)	- Effect of sociodemographics and health status	- Mixed logit models<br>- Latent class analysis	➢ **Health status**: patients in better health → higher dis-preference for severe levels of pain or FWB<br>➢ Four classes<br>○ **Class 1: prefer to receive cancer treatment** - dis-preference for OOP, hyperglycemia, rash and severe levels of pain and impairment of FWB<br>○ **Class 2: treatment avoiders** - important attributes: OOP, hyperglycemia, and severe pain<br>○ **Class 3**: important attributes: severe pain and severe impairment FWB<br>○ **Class 4**: makes counterintuitive choices – higher utility from paying OOP for their treatment

Simes et al., 2001	- Primary analyses were nonparametric	➢ A large majority of the patients felt that **relatively modest improvements in survival duration or in the percentage chance of 5-year** survival **would justify 6 months of the treatment** they received<br>➢ For some women **even very large survival benefits would be insufficient to justify the toxicity** of treatment.	- Patient and disease factors predicting individual preferences	- Kruskal–Wallis test (comparison of preferences for major groups)<br><br>- Multivariate linear regression analysis (patient and disease factors predicting individual preferences)	➢ **Support needed by dependents:** Patients with dependents requiring full or partial support → more likely to find treatment acceptable and needed smaller survival gains to justify treatment<br>➢ **Support available to patient:** Patients to whom full support was available from others → accepted smaller increments in survival as justifying treatment<br>➢ **Treatment related toxicity:** Patients experiencing worse toxicity → demanded greater improvements in survival to justify treatment<br>➢ **Dosage reduction during chemotherapy:** Patients whose chemotherapy dosage was reduced to 75% or less of the total planned dose → demanded longer survival increments<br>➢ **Initial radiotherapy:** Patients whose initial adjuvant treatment included radiotherapy as well as chemotherapy → required larger increments of survival to justify treatment<br><br>*Age, education level, employment status, time between treatment and interview, use of concurrent adjuvant endocrine therapy, occurrence of relapse, use of any particular modality for the treatment of relapse*: No associations were observed
Galper et al., 2000	- Descriptive statistics – characterize the responses to each hypothetical scenario obtained	➢ Substantial improvements in local control of the cancer and overall survival required before opting for the procedure	- Determine the impact of **sociodemographic**, **clinical,** and **psychosocial factors** and **the presence of perceived or actual upper-arm dysfunction** on preferences for axillary lymph node dissection (ALND)	- Univariate analyses	➢ None of the clinical, sociodemographic, or psychosocial factors consistently explained more than 5% of the variability in preferences across all four scenarios
Stamuli et al., 2022	- Conditional logit model	➢ Most important elements driving patients' choice of treatment:<br>○ ability to function well in daily life<br>○ avoid severe levels of pain<br>➢ Willingness to trade off months of PFS to:<br>○ Move from exterme impairment in functional well being (FWB) to no impairment<br>○ Move from pain to no pain	- Patients' preferences by **country**<br><br>- Patients' preferences by **socio-demographic** and **disease characteristics**	- Conditional logit model (individual country analyses)<br><br>- Subgroup analyses	➢ **Age:** Older respondents (> 54) → value 'no pain' and both levels of FWB attributes more than younger patients<br>➢ **Level of education:** Educated patients → higher value on PFS<br>➢ **Stage of cancer:** Patients with advanced stage of the disease or in remission → higher value on levels of pain and FWB
Mansfield et al., 2023	- Error component (EC) random-parameters logit (RPL) model	➢ Most importance: change in PFS from 5 to 26 months<br>➢ Largest minimum acceptable benefit (MAB) required in additional months of PFS: 1) accepting a 15% risk of heart failure, 2) moving from no risk of serious lung damage and infections to 15% risk, 3) moving from no liver function problems to possible severe liver function problems	- Subgroups defined by cancer stage, prior breast cancer treatment experience, HER2 status, whether respondents had children, employment in the medical field, and the respondent's country of residence	- Subgroup analyses (subgroup models estimated using the EC RPL model)	➢ **HER2 status:** additional months of PFS were more valued by HER2-negative respondents compared to other attributes, in contrast to HER2-positive respondents<br><br>➢ **Presence of children**:<br>○ with children → greater emphasis on increasing PFS<br>○ without children → stronger preference for avoiding liver function issues, diarrhoea, and nausea/vomiting relative to other attributes

					➢ **Country of residence:**<br>○ Respondents in the US and UK valued increasing PFS more than respondents in Japan<br>○ Respondents in Japan showed a stronger preference for avoiding heart failure, liver function problems, and serious lung damage or infections compared to those in the US and UK
McQuellon et al., 1995	- Descriptive and univariate statistics	➢ Greater toxicity potential → less likely to accept treatment<br>➢ Almost all patients would accept treatments for a 5 year increase in survival	- Associations between continuous variables and the patients' willingness to accept chemotherapy treatment	- Fisher's exact test	➢ **Age:** Younger patients → more willing to assume the risks of treatment for a small increase in life expectancy<br>➢ **Prior therapy**:<br>○ Subjects who had received adjuvant chemotherapy → more likely to prefer not waiting until their cancer caused symptoms before starting treatment in both the standard and experimental chemotherapy scenarios & more inclined to choose standard chemotherapy over hormonal therapy<br>○ Currently on hormone treatment → less likely to opt for high-dose chemotherapy compared to standard chemotherapy<br>○ Subjects with prior radiation treatment → more likely to choose standard chemotherapy over hormonal therapy
Spaich et al., 2019	- Trade-off technique; quantify the additional risk of recurrence that patients would be willing to accept with each treatment modality	➢ 42.5% of patients would accept additional risk of recurrence for intraoperative radiotherapy (IORT) **versus** 9% for external beam radiotherapy (EBRT)	- Socioeconomic factors, demographic factors and general attitutes with an influence of the additional accepted risk	- Multivariate regression model	➢ No significant associations were found for any of the following parameters
Reinisch et al., 2021	- Hierarchical bayes approach; individual choices calculated via a logit choice model – group behavior using a multivariable normal distribution	➢ *Highest utility:* 1) QoL attribute 'physical agility and mobility' 2) treatment goals (OS & PFS) 3) therapy related side effects<br>➢ *Most important side effect with regard to treatment decisions*: nauseau/vomiting<br>➢ *Side effects most frequently wished to avoid*: 1) hair loss, 2) nausea and vomiting, 3) fatigue, 4) infection	- Differences between first and second line patients	- Gibbs sampling procedure	➢ OS and PFS slightly more important to patient receiving second line vs. first line treatment<br>➢ Hair loss was significantly more important to first-line patients
Ngorsurach es et al., 2015	- Multinomial logit model	➢ Importance weighted on PFS, anemia and pneumonitis<br>➢ Pneumonitis & anemia:<br>○ Pneumonitis risk weighted higher than anemia risk<br>○ Accept higher risk for anemia than for pneumonitis to trade for PFS	/	/	/
Duric et al., 2005	- Preferences are presented descriptively with figures and frequency tables	➢ Preferences were highly variable<br>➢ Improvements of **an additional year in life expectancy or 3% in survival rates** were judged sufficient to make adjuvant	- Associations between patients' characteristics and preferences	- Linear regression	➢ Women's baseline characteristics and preferences: not strongly associated<br>➢ Women's recollections of their experience of adjuvant chemotherapy and preferences: strongly associated

		chemotherapy worthwhile by 68–84% of women			○ Women who were more troubled by adjuvant chemotherapy → larger benefits necessary
Duric et al., 2005	- Preference data described with grahs of the proportions of women judging various benefits	➢ Preferences were highly variable<br>➢ About a third of women judged **gains of 1% in survival rate or 6 months in survival time sufficient** to make adjuvant endocrine therapy worthwhile	- Association between preferences and baseline characteristics	- Linear regression	➢ Two factors significantly associated with preferences:<br>○ Women with few side effects → smaller benefits judged sufficient<br>○ Women treated with Tamoxifen → smaller benefits judged sufficient
Omori et al., 2019	- Conditional logit model	➢ Strong preference for treatment that **extend progression free survival (PFS)**<br>○ *Frequency of stools (FOS) was 6 (grade 2 diarrhoea)* → order of attributes' relative importance: PFS, DOD (duration of diarrhoea), FOS, IOD (incidence of diarrhoea) and RFA (route and frequency of administration)<br>○ *FOS was 9 (grade 3 diarrhoea)* → FOS most important attribute	- Based on sociodemographic characteristics and clinical characteristics	- Conditional logit model including patient-specific covariants<br>- Subgroup analyses	➢ **Patient-specific covariates – no statistical significance but:**<br>○ a relapse or metastases → strongest preference for the longest PFS<br>○ aged between 45 and 59 years → weakest preference for the highest FOS (grade 3 diarrhoea)<br>➢ **Subgroup analyses**:<br>○ *experience with relapse and/or metastasis* & *hormone resistance or no experience of endocrine therapy & aged between 45 and 59 years & married respondents & whose youngest child was aged < 20 years & who had no children* → FOS was 6 (grade 2 diarrhoea), the longest PFS (16 months) was the most important attribute
Nazari et al., 2021	- Conditional logit regression model	➢ Ranking of relative attribute importance: (1) effectiveness (PFS); (2) cost; (3) neutropenia; (4) stomatitis; (5) arthralgia; (6) administration	- Based on disease stage (metastatic or nonmetastatic) and on menopausal status (postmenopause or premenopause)	- Exploratory subgroup analyses	➢ **Cancer stage**:<br>○ patients with non-metastatic cancer derive more benefit from longer PFS and are less concerned about avoiding side effects<br>➢ **Menopausal status**:<br>○ premenopausal breast cancer patients gain less benefit from avoiding risks (adverse events)<br>○ postmenopausal patients are less willing to pay for higher levels of monthly costs
Kuchuk et al., 2013	- Utility scores for a health state (probability at the indifference point between choosing to stay in the respective health states vs. taking a gamble)	➢ Lowest utility (i.e., least preferred): **grade III/IV nausea/vomiting**, **grade III/IV diarrhoea** and **grade III/IV sensory neuropathy**<br>➢ Worst side effect indicated by most patients: **grade III/IV nausea/vomiting**	- Cancer stage, age and marital stage	- Exploratory subgroup analyses	➢ No statistically significant differences were observed in the **standard gamble utilities** by cancer stage, age and marital stage<br>➢ Responses to the agree/disagree statements regarding the **importance of survival, control of tumor growth**, and **decreasing quality of life** *(not significant)*<br>○ Patients in earlier stage → higher importance on survival<br>○ Patients in more advanced stage → greater importance on QoL
Williams et al., 2021	- Conditional logit model	➢ Treatment choice most affected by preferences related to: i) treatment affordability, ii) impact of treatment on activities of daily living, iii) being a burden on friends, family or care partners, iv) physical side effects of treatments	- Two archetypes characterized by preferences regarding breast cancer treatment	- Latent class analysis	➢ Two distinct treatment preference archetypes emerged:<br>○ **Cost-prioririzing group:** most concerned about affordability, impact on activities of daily life, and burdening care partners

					○ **Functional independence–prioritizing group:** concerned about their ability to work, physical side effects, and interference with life events
Thill et al., 2016	- Analytical hierarchy process methods	➢ Most important therapeutic target: achievement of pathological complete remission (pCR)<br>➢ Least important: avoidance of side effects<br>○ In the side effects, most important fatigue	/	/	/
Wouters et al., 2013	- Average and spread of utilities and relative importance percentages	➢ Attributes with the highest relative importance: osteoporosis, efficacy, risk of endometrial cancer, joint and muscle pain	- Association between benefit/drawback ratio and demorgraphic and clinical characteristics	- Linear regression analysis	➢ Benefit/drawback ratio and …<br>○ **higher educational level** & **past treatment with cytostatic therapy**: positively associated<br>○ **age**: negatively associated
Bullen et al., 2024	- Error component logit model – relative importance of attributes & minimum acceptable survival for improvements in side effects	➢ Largest relative importance: 1) overall survival, 2) other side effects, 3) diarrhoea<br>➢ For all grade 2 side effects – willing to forgo some absolute probability of overall survival	- Comparing patients with metastatic breast cancer and patients with primary breast cancer	- Comparing estimated importance	➢ The most notable difference: the estimated importance of the nausea attribute<br>○ There are no statistically significant differences between any of the estimates
Beusterien et al., 2014	- Conjoint analysis	➢ Among grade I/II side effects largest impact on preferences: a 5% reduction in the risk of 1) sensory neuropathy, 2) nausea, and 3) motor neuropathy<br>➢ Among grade III/IV side effects largest impact on preferences: a 5% reduction in the risk of 1) motor neuropathy, 2) nausea, and 3) myalgia<br>➢ Oral twice-daily regimen: most preferred	/	/	/
Thewes et al., 2005	- Proportion of women accepting endocrine therapy for various gains in survival were analysed with descriptive statistics	➢ Half the participants judged that a 2% gain in probability of survival was sufficient to make endocrine therapy worthwhile<br>➢ Half the women judged that a gain of 3 months was sufficient to make adjuvant endocrine therapy worthwhile if their life expectancy without it was 5 years	- Associations between predictor variables and preferences	- Linear regression	➢ Total time benefit (TTB) was significantly associated with the severity of endocrine therapy side effects
Lalla et al., 2014	➢ Conjoint analysis process – obtain utilities	➢ Attributes with the most utility to patients: 1) risk of infection, 2) diarrhoea, 3) nausea<br>➢ Side effect patients were willing to pay the most to avoid: 1) severe diarrhoea, 2) being hospitalized due to infection, 3) severe nausea	/	/	/
