# Supplementary material for: Patient Preferences in Breast Cancer: A Scoping Review
Source: Cancers (Basel). 2025 Dec 31;18(1):134. doi: 10.3390/cancers18010134 (PMC12784654; doi:10.3390/cancers18010134)
Supplement: Supplementary file 1 [file cancers-18-00134-s001.zip › Table S8. Evaluation and future research.pdf]

## Table S8: Evaluation and future research

| Study | Limitations | Strengths | Opportunities - Future research |
|---|---|---|---|
| Tan et al., 2014 | - **small sample size**<br>- to reduce respondent burden - only 14 of the 22 health states under evaluation<br>- only outpatients - **selection bias** - patient with better health and mobility<br>- ping-pong approach not used in standard gamble (SG) assessment<br>- 50% prefect health starting point - narrower ranges of preferences - **starting point bias** | - visual analogue scale (VAS) and SG methods - practical, reliable and valid<br>- high completion rate | - info for **future cost-utility analysis** of breast cancer treatments<br>- may be used as a basis to obtain utility data from other Asian populations |
| Srikanthan et al., 2019 | - **small sample size** - prevented analyses from identifying predictors for trade-offs<br>- **selection bias** - all patients underwent chemotherapy<br>- baseline fertility – may have impacted trade-off preferences<br>- no inclusion of newly diagnosed patients | - **in person interviews** - clarify if the scenarios were well understood<br>- **mimicked the physician - patient encouter →** increasing generalizability of the results to real world scenarios<br>- 92% of the patients found the **treshold task and the information presented easy to understand** | - **information** on chemotherapy-associated infertility; provision of written materials, web-based decision aids; **promote discussion and documentation**<br>- multi-disciplinary counseling |
| Silva et al., 2022 | - **small sample** - compared to the population in Brazil - not a representative sample<br>- data collection instrument - **not a validated questionnaire** | / | - importance of **health-quality of life**<br>- improvements in the **sensitivity of instruments** capturing issues related to quality of life<br>- taking into account the **experience of patients**; improvements in the process of health technologies assessment are patient-centered |
| Ballinger et al., 2017 | - inherent **weakness of the choice-based conjoint (CBC) analysis**: impossible to know if the same choice woud be made in an actual therapeutic decision<br>- missing preferences of patients who have not yet undergone chemotherapy<br>- biases by **individual experiences**; may be an over-estimation by the patients of their risks | / | - patients consider **many variables** when making treatment decisions<br>- decision-making is **affected by information** provided<br>- future studies of biomarker significance including measures of clinical relevance |
| Wouters et al., 2013 | - sample non-adherence not as pronounced - **selection bias**<br>- number of statements limited - selection bias | - combination of **in depth online focus groups** & the **Q-sorting task**<br>- large number of women from various backgrounds<br>- discussion of a large number of themes | - women's own perceptions and experiences about endocrine therapy targeted to improve adherence |
| Hollin et al., 2020 | - stated choices between **hypothetical treatments** vs. real world decisions<br>- number of attributes - greater **burden on respondents** | / | - value frameworks important step in systematic evaluation of medication; DCEs can offer information to be used on the |

| | | | |
|---|---|---|---|
| | - rely on **self-reporting** of diagnosis and disease stage<br>- **small and homogeneous sample size** - not possible to establish the generalizability of the results | | **patient-centric scoring rubric for value frameworks**, and to determine the **apporpriate number of clinical screnarios** |
| Smith et al., 2014 | - **not representative** of all women with breast cancer<br>- **many variables** not considered<br>- statistical analyses were **not controlled for multiple comparisons** | / | - future research on a more representative population, other settings, other drug profiles |
| Chou et al., 2020 | - **selection bias** - convenience sampling<br>- health states drafted **without patient input**<br>- **multiple methods and time horizons** utilized - could make comprehension challenging | / | - utility values provide information for future cost-utility analyses |
| DaCosta DiBonaventura et al., 2014 | - **selfreported data** on treatments and adherence information - **recall biases and self-presentation effects**<br>- **simplifying hypothetical exercise** - may differ with the inclusion of other attributes<br>- **different analytical methods** - difficult to directly compare preference results<br>- **detailed information** on disease progression, treatment chronology, side effects experienced, and dosing adjustments was **unavailable** → context for the results on adherence is incomplete<br>- **convenience sample** | / | - **greater education about potential side effects and memory aids**; further research to understand these potential interventions and their effectiveness in this patient population<br>- further research to **enhance the understanding of the factors influencing adherence rates** in the treatment of breast cancer, and to develop effective interventions in line with advances in the pharmaceutical industry |
| Liu et al., 2024 | - may be **difficulties understanding attributes** infections and thrombosis risk<br>- **limited attributes**<br>- **unlabeled design** may have introduced biases<br>- **efficient design approach**<br>- **patient sample** - recruited from three major hospitals located in developed and affluent cities in China - may **not be representative** of patients across China as a whole | / | / |
| Stamuli et al., 2023 | / | / | - **patient preferences**, in combination with clinical guidelines → **support the selection and tailoring of treatment options**<br>- innovative reimbursement decision making approaches **prioritizing a patient-centered perspective**, by considering the diverse needs and preferences of different patient groups<br>- **engaging with decision makers** and stakeholders is important to ensure that patient-preference studies are relevant, useful, and impactful in real-world decision making |
| Simes et al., 2001 | - **only the benefit survival gains** was assessed<br>- **no phsychological benefits** taken into account<br>- **sample representation**<br>- no information what would happen to survivors beyond 5 years | - **consistency of preferences over time** - presumably related to the use of standardized interviews<br>- use of **woman's experiences of adjuvant chemotherapy** | - additional studies: guide the use of more recently developed adjuvant chemotherapy regimens |

| | | | |
|---|---|---|---|
| Galper et al., 2000 | - **study population**: choice of two groups providing useful insights but that this would not interfere with their care - homogenous - in a single network of affiliated academic hospitals - not representative for all breast cancer patients | / | / |
| Stamuli et al., 2022 | - **attribute out of pocket payments for treatments** - many patients may not regularly experience this - limitation when using individual values of marginal rates of substitution (MRS)<br>- presentation of the choice evaluation and framing of the DCE question → omitted attribute bias<br>- interactions between attributes can not be determined<br>- data collection - **online panel** - bias towards patients that are technology savvy and in better health states<br>- **patients in different health states** - some patients might have related to the profiles better and actual experience | - **absence of specific treatment descriptors** → findings are **usable for every viewpoint** (regulatory, HTA, patients and physicians) | - patient preferences - may **enhance the medical, regulatory and HTA decision-making process** |
| Mansfield et al., 2023 | - **data was self-reported**<br>- treatment features based on HER2-targeted treatement for metastatic breast cancer **vs**. sample with women stage III and IV breast cancer with any HER2 status<br>- **attribute choice PFS** instead of the golden standard OS<br>- **limited number of attributes** can be included - other factors not included can still have an influence<br>- **missing demographic data** - subgroups incomplete<br>- **hypothetical scenarios**<br>- **generalizability of results** to other countries & early stage breast cancer<br>- online setting<br>- influence of COVID-19 for the results from Japan | / | / |
| McQuellon et al., 1995 | / | / | / |
| Spaich et al., 2019 | - **older patient population** → might limit the finding for younger breast cancer patients<br>- influence of the **baseline risk of recurrence**<br>- larger cohort would help to detect significant combinations of factors that drive preferences | / | - **improve shared decision-making** and individualised approaches in breast cancer therapy → it appears paramount to identify factors that drive patient preferences and to thoroughly **characterise respective subgroups** |
| Reinisch et al., 2021 | - **attributres could not be deselected**<br>- voluntary particpation; **participant self-selection** | / | - **important to consider** quality of life (**QoL**), overall survival (**OS**) and progresion-free survival (**PFS**) in treatment decisions with patients with advanced breast cancer (aBC) |

| | | | |
|---|---|---|---|
| Ngorsuraches et al., 2015 | - **small sample size** → association between income and willingness-to-pay (WTP) not possible to examine<br>- does **not reveal true preferences**, decisions are not really made<br>- **limited number of attributes** - other attributes could also affect preferences<br>- attributes of a specific treatment to rank **vs.** study population (any stage of breast cancer)<br>- patients from only one hospital in southern Thailand | / | - results of the study could **help clinicians in choosing the treatments** based on patients' preferences |
| Duric et al., 2005 | - limitations on **sampling frame** and **generalisability** | / | - Explicitly considering **benefits and harms** in chemotherapy discussions<br>- Different group of people → **different preferences for chemotherapy**<br>- Reconciling the **range and diversity of perspectives**<br>- Understanding **factors that might influence decision-making**<br>- **Clinicians' recommendations** → also reflect the particular patient's attitudes, values and priorities |
| Duric et al., 2005 | - representation of the sample<br>- preferences based on women's recollections of what treatment was like | / | - future studies: **explore importance of fertility** related issues to younger women concerning adjuvant endocrine therapy<br>- **preference information, benefits of treatments required** - valuable for clinicians, patients and communities<br>- future research: **best way to elicit and incorporate this information** in clinical decision-making |
| Omori et al., 2019 | - **generalizability** of the results<br>- **online survey** - biased towards participants confident in the use of technology and had access<br>- **limited number of attributes** - other attributes may also affect patients' preferences | - use of a **large database**<br>- **pilot phase** of the study - allowed improvements | - **importance of preventing and managing diarrhoea** → maintain patients' motivation for the treatments<br>- patients' sociodemographic and clinical characteristics → affects patients' treatment choices → help physicians to develop **strategies of treatment choice** |
| Nazari et al., 2021 | - **difficulties in recruiting patients**<br>- choices made in an **hypothetical environement** may be different in actual real life<br>- **small, convenient sample** - patient willing and healthy enough to participate<br>- limitations of the conditional logit model | / | - information can be used by **treating physicians in the treatment strategy**; by **health authorities** for submitted dossiers; for **pricing and market access** negotiations<br>- future researchers to **consider the type of insurance** of the patients in their analysis |
| Kuchuk et al., 2013 | - **descriptions may not be representative** for the experiences of patients<br>- idea of assigning **a particular health state "for the rest of your life"**<br>- **web survey** → may have contributed to the high illogical or invariable responses obtained<br>- **patient selection bias** – patient had to be comfortable using a computer<br>- **survey fatigue**; patients being less careful about reponses | / | - **utility weights** could be used **in future cost effectiveness evaluations** → quality adjusted life expectancy based on the patient prespective |

| | | | |
|---|---|---|---|
| Williams et al., 2021 | - **not representative** for all breast cancer patients<br>- **not all preferences** may be included in the survey<br>- all **data was self reported** | / | - consideration and evaluation of patient preferences related to both **the direct and indirect costs of treatment** during treatment decision making<br>- patient focussed drug development |
| Thill et al., 2016 | - data exclusively from patient interviews | - comprehensive interviews conducted by experienced interviewers | / |
| Wouters et al., 2013 | - adaptive conjoint analysis (ACA) task – relative risk reduction could have been much higher then in reality<br>- ACA task – might be difficult to understand<br>- Overrepresentation of the higer educated | - use of an ACA choice task - mimic the trade-offs in the real world for patients | - identify women at risk for nonadherence → help clinicians in tailoring the communication; reassure women who are concerned<br>- examine wheter the preferences change over time |
| Bullen et al., 2024 | - **sample size**, age in the sample<br>- preferences can be **influenced by many factors**<br>- descriptions of side effect – may be difficult to be understood<br>- choice task – simplified to **only one risk attribute**<br>- **interpretation of the no treatment option** may have been different for different respondents<br>- **recruitment difficulties** | - DCE methodology – trade-offs can be estimed in accordance with economic utility theory<br>- selection of attributes informed by qualitative methods and literature | - inform person-centred care and shared decision-making<br>- further incorporation of patient-reported outcomes (PROs) and preference studies in the study of new medicines for mBC<br>- future research is to compare the preferences of patients and doctors |
| Beusterien et al., 2014 | - descriptions many not be representative<br>- **patient selection bias**<br>- **lenghty survey** – grown fatigue, less careful about the responses | / | - understanding and incorporating preferences into treatment plans for breast cancer patients → improved clinical and quality of life outcomes |
| Thewes et al., 2005 | - generalizability of the results<br>- bias: cognitive dissonace reduction: make current attitudes and beliefs based on previous decisions<br>- cross-sectional study: stability of preferences over time not explored<br>- study was exploratory → any association may be due to chance<br>- multicentre study: participants were seen by different clinicians – type of information probably varied | / | - **Future research**: stability of preferences for endocrine therapy over time; what is needed to make the therapy worthwile; explore the method of information provision - and the relationship with the needed information<br>- importance of **discussing risks, benefits, harms, and priorities** with patients |
| Lalla et al., 2014 | - results can be due to **varies determinants**<br>- results of the study **not generalizable** for the whole metastatic breast cancer population | / | - understanding patients' perspectives and preferences on side effects they wish to avoid most → value based decision making in selecting treatments |
